# Enhanced Microwave Absorption Performance of Amorphous Co_100−x_Fe_x_ Nanoparticles

**DOI:** 10.3390/nano15141091

**Published:** 2025-07-14

**Authors:** Zhen Wang, Chao An, Fenglong Wang, Hongsheng Liang, Zhaoyang Hou, Hao Shen, Hongjing Wu

**Affiliations:** 1 Department of Applied Physics, School of Science, Chang’an University, Xi’an 710064, China; wangfenglong@chd.edu.cn (F.W.); hongshengliang@chd.edu.cn (H.L.); houzy@chd.edu.cn (Z.H.); 2 School of Intelligent Manufacturing Engineering, Chongqing University of Arts and Sciences, Chongqing 402160, China; anchao@cqwu.edu.cn; 3 MOE Key Laboratory of Material Physics and Chemistry Under Extraordinary, School of Physical Science and Technology, Northwestern Polytechnical University, Xi’an 710072, China; wuhongjing@nwpu.edu.cn

**Keywords:** amorphous Co_100−x_Fe_x_ nanoparticles, complex permittivity and permeability, microwave absorption properties

## Abstract

Metallic magnetic materials are extensively used to mitigate electromagnetic interference due to their high Curie temperatures and permeability. However, their high permittivity often hinders impedance-matching effectiveness, limiting their utility. In this study, amorphous cobalt–iron (Co_100−x_Fe_x_) alloy nanoparticles with relatively low permittivity were synthesized using a simple aqueous reduction method at room temperature. The effect of atomic ratio variation on the microwave absorption properties of these nanoparticles was investigated across 2–18 GHz. The amorphous Co_100−x_Fe_x_ nanoparticles exhibited excellent electromagnetic wave absorption performance, achieving an effective absorption bandwidth of 5.6 GHz, a matching thickness of 2.60 mm, and a reflection loss of −42 dB.

## 1. Introduction

The electromagnetic (EM) wave radiation generated by wireless communication and computer technology has evolved into serious pollution that harms human health and interferes with the operation of high-precision electronic apparatus [1,2,3]. Consequently, microwave absorption materials have attracted significant interest in regard to addressing these issues. In the EM absorption process, EM energy is converted into other forms of energy, which depends on the inherent properties of the materials (such as carrier transport capacity, dipoles, interfaces, defects, and magneto anisotropy) and their macroscopic structures. In order to reach excellent absorption property, first, the impedance matching between the material and the free space demands that the permittivity should be equal to the permeability of materials. Second, the absorption material should have large imaginary permeability and/or permittivity within the microwave frequency range to attenuate the incident electromagnetic wave [4,5,6,7,8].

CoFe-based alloys have attracted significant interest due to their elevated magnetizations, along with high Curie temperatures, and their complex permeability values can remain at high levels in the gigahertz range compared with oxide [9,10], such as magnetic tunnel junctions (MTJs), anisotropic magnetoresistance, and so on [11,12,13]. However, metallic magnetic materials always show high permittivity, which hinders impedance matching and limits their applications [7,14]. In order to reduce the permittivity in metallic magnetic materials, the core/shell structure has been proved to be a feasible method for achieving excellent EM absorption properties [15,16,17,18,19]. Changing the morphology of metallic magnetic materials also can obtain relatively low permittivity [20,21,22,23,24]. Meanwhile, low permittivity can be found in some amorphous metallic alloys as compared with crystalline metals [14].

Thus, in this paper, amorphous Co_100-x_Fe_x_-alloy nanoparticles with different atomic ratios were synthesized by aqueous reduction using NaBH_4_ at room temperature [10]. Amorphous metallic alloys, due to their lack of long-range crystalline order, show unique electronic, magnetic, and corrosion-resistant properties [25,26], which may play an important role in the practical application of EM absorption. The morphology, magnetic, high-frequency complex permittivity, and permeability characteristics, as well as the microwave absorption capability of amorphous Co_100−x_Fe_x_-alloy nanoparticles, have been investigated.

## 2. Materials and Methods

### 2.1. Synthesis of the Amorphous Co_100−x_Fe_x_ Nanoparticles

Amorphous Co_100−x_Fe_x_ nanoparticles were synthesized by aqueous reduction in iron II sulfate (FeSO_4_·7H_2_O, 99.9%, Beijing Chemical Reagent Company, Beijing, China) and cobalt sulphate (CoSO_4_·7H_2_O, 99.9%, Fengshun Fine Chemicals Company, Meizhou, China) by using sodium borohydride (NaBH_4_, 98%, Aladdin Scientific Corporation, Riverside, CA, USA) and sodium citrate (Na_3_C_6_H_5_O_7_·2 H_2_O, 99%, Aladdin Scientific Corporation, Riverside, CA, USA) [27]. In the experimental process, a borohydride-to-metal ratio and a metal-to-citrate ratio were kept at 2:1 and 10:1, respectively. In the experiment, 4.6 mM sulphate of metal (the molar ratio of CoSO_4_·7H_2_O:FeSO_4_·7H_2_O with 1:0, 7:3, 5:5 3:7 and 0:1) and 0.46 mM trisodium citrate dihydrate were mixed vigorously with a magnetic stir bar in 2 L of deionized water until dissolved entirely. A total of 8.80 mM sodium borohydride was prepared and then was added to the mixture and allowed to react for about 30 min. The precipitate was magnetically separated and washed several times with ethanol and deionized water. After washing, the precipitate was placed in a vacuum environment to dry overnight prior to analysis. Then, the amorphous Co_100−x_Fe_x_ nanoparticles were prepared for measurements.

### 2.2. Characterization of the Amorphous Co_100−x_Fe_x_ Nanoparticles

The crystal phase analysis of Co_100−x_Fe_x_ was performed by powder X-ray diffraction (XRD) equipment on a D/Max-2400 (Rigaku, Japan) with Cu Kα radiation. Morphology observations of the nanoparticles and the elemental compositions were conducted by scanning electron microscopy (SEM) and energy-dispersive X-ray spectrometer (EDX) in 6610 (JEOL, Japan). The elemental ratio was also determined by the inductively coupled plasma (ICP) approach (Optima 5300 DV, USA). X-Ray photoelectron spectroscopy (XPS) survey scans were used to determine the elemental surface chemistry by a Kratos Axis Ultra DLD photoelectron spectrometer (Japan) with a monochromatic Al Ka (1486.6 eV) and Mg (1256.4 eV) source at 300 W (10 mA × 15 kV) and a pass energy of 20 eV for high-resolution scans. The static magnetic properties of the samples were estimated by a Lakeshore 7304 vibrating sample magnetometer (VSM, USA). The complex relative permeability ($\mu_{r}=\mu^{\prime }-j\mu \prime \prime$) and permittivity ($\varepsilon_{r}=\varepsilon^{\prime }-j\varepsilon \prime \prime$) spectra of the composite samples were measured by the coaxial method on an Anritsu MS46322B vector network analyzer (VNA, Japan) using the transmission/reflection mode within the range of 2–18 GHz [28]. For the VNA measurement, the samples were dispersed in paraffin wax homogeneously with a sample-to-paraffin wax weight ratio of 1:1 by an ultrasound process. Subsequently, a toroidal die assembly was used to press the mixture into a toroidal shape with an inner diameter of 3.04 mm and an outer diameter of 7.00 mm, which was prepared to fit well with the coaxial sample holder for microwave measurement. It should be noted that the complex permittivity and permeability of wax are constants within the whole measurement frequency range: $\varepsilon_{r}=2.2-j_{0}$, $\mu_{r}=1.0-j_{0}$. Then, the reflection loss and reflection loss factors were calculated by the complex permittivity and permeability.

## 3. Results and Discussion

### 3.1. Morphological and Structural Characterization of the Amorphous Co_100−x_Fe_x_ Nanoparticles

**Figure 1:**
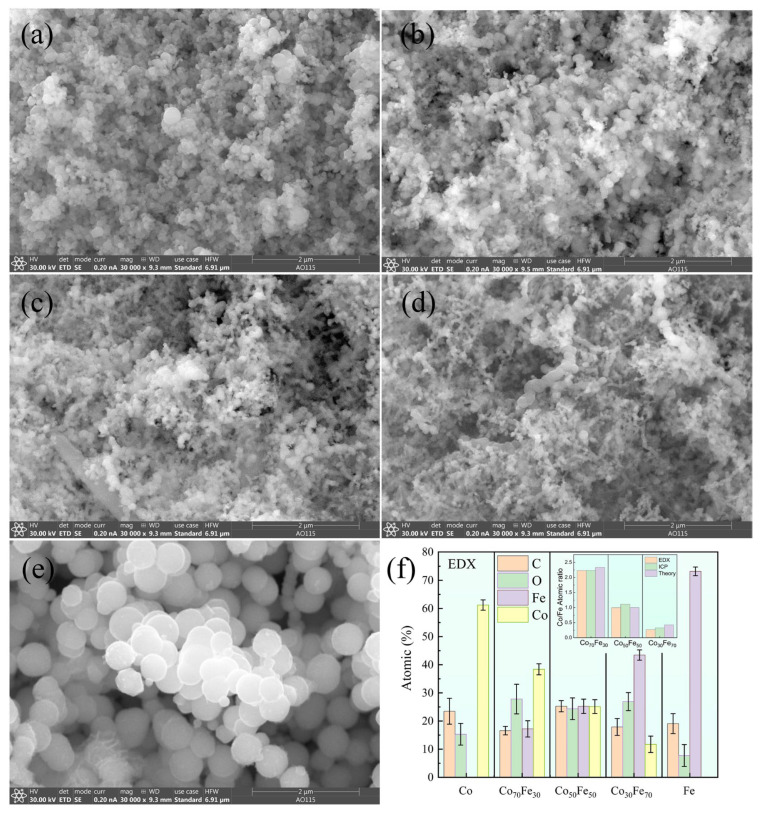
SEM images of Co (**a**), Co_70_Fe_30_ (**b**), Co_50_Fe_50_ (**c**), Co_30_Fe_70_ (**d**), and Fe (**e**). EDX results (**f)**. The insert is the Co/Fe atomic ratio from the EDX, ICP, and theory.

The morphology features of amorphous Co_100−x_Fe_x_ nanoparticles were characterized by SEM in Figure 1a–e. As shown, all samples exhibit spherical morphology. Co_100−x_Fe_x_ particles with *x* = 0, 30, 50, and 70 demonstrate similar morphologies, with diameters from 10 to 100 nm and an average diameter of approximately 75 nm. However, the Fe particles (*x* = 100) show a significantly larger diameter of about 430 nm as compared with other compositions, as shown in Figure 1e, which clearly indicates that the presence of Co effectively refines the particle morphology of Fe. EDX analysis in Figure 1f reveals the presence of C and O in addition to Co and Fe. The carbon content may originate from the trisodium citrate used in synthesis and possible atmospheric carbon adsorption during air exposure. The oxygen content likely results from the surface oxidation layers formed when the nanoparticles were exposed to ambient air. The inset in Figure 1f compares the atomic ratios of Co and Fe obtained from the EDX, ICP analysis, and theoretical values [29]. The EDX and ICP (Table S1, Supporting Information) results show reasonable error values, which indicates that measurement results are highly reliable. Both the EDX and ICP results show good agreement with the initial molar ratios of the Co and Fe precursors used in synthesis.

**Figure 2:**
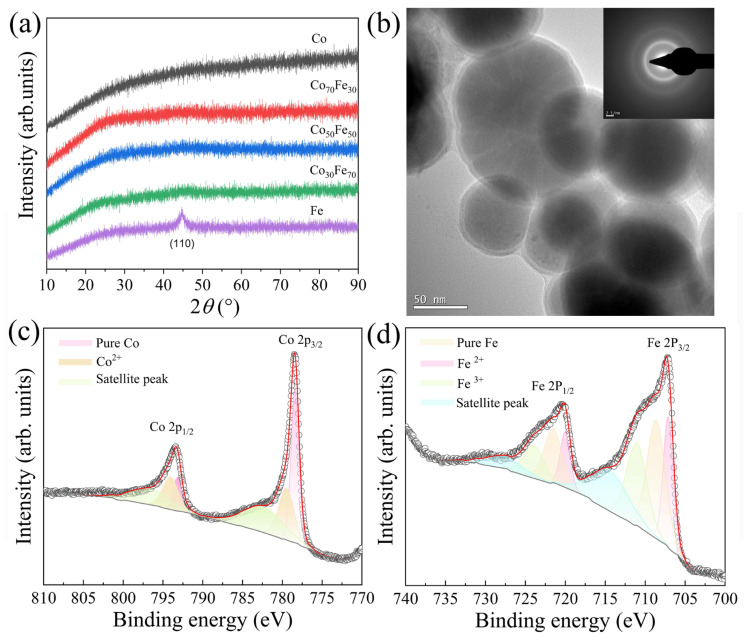
XRD patterns of Co_100−x_Fe_x_ (**a**). TEM of Co_50_Fe_50_, and the insert is the selected area diffraction pattern of Co_50_Fe_50_ (**b**). XPS high-resolution scans spectra of Co (**c**) and Fe (**d**) in Co_50_Fe_50_.

All Co_100−x_Fe_x_ samples exhibit an amorphous phase, as shown by the XRD patterns in Figure 2a. A weak diffraction peak at approximately 44.5° was observed for the Fe nanoparticles, corresponding to the (110) crystallographic plane of crystalline Fe (BCC structure). The amorphous nature of these alloys likely influences their electronic structures and magnetic properties. Figure 2b shows the TEM image of the Co_50_Fe_50_ nanoparticles, revealing a particle diameter of approximately 100 nm, consistent with the SEM results. The inset in Figure 2b displays the selected area electron diffraction (SAED) pattern, which confirms the amorphous nature of the Co_50_Fe_50_ alloy. Figure 2c,d presents the high-resolution XPS spectra of Co and Fe in the Co_50_Fe_50_, respectively. For the Co 2p region (2p_3/2_ and 2p_1/2_ spin-orbit doublet), multiple peaks are clearly resolved. Peak deconvolution reveals three distinct components at binding energies of 778.0 eV (2p_3/2_)/793.0 eV (2p_1/2_), 779.3 eV (2p_3/2_)/794.0 eV (2p_1/2_), and 782.6 eV (2p_3/2_)/796.1 eV (2p_1/2_), corresponding to metallic Co, Co^2+^ species and a shake-up satellite peak, respectively [30,31,32]. The area ratio of Co metal to Co^2+^ to Co satellite is 30.5:32.3:37.2 in the curve, which indicates that the Co has been oxidized. For Fe 2p_3/2_ (2p_1/2_), the peak can be fitted with four components. From low to high binding energy with a peak at 707.1 (720.0), the eV corresponds to pure Fe, 708.6 (721.5) and 711.1 (723.9) eV indicate the existence of Fe^2+^ and Fe^3+^ in the interface, while 714.5 (727.7) eV is the satellite peak [31,32]. The area ratio of Fe metal, Fe^2+^, Fe^3+^, and the Fe satellite peak is 31.2:35.8:23.3:9.7, indicating that Fe has reacted with organic species. When metal nanoparticles are exposed to air, a thin oxidation layer inevitably forms on their surfaces. Consequently, the oxygen signal observed in the XPS results originates from the surface oxides, which is consistent with the EDX results. This surface oxidation is also one of the factors contributing to the reduction in dielectric properties.

### 3.2. Static Magnetic Characterization of the Amorphous Co_100−x_Fe_x_ Nanoparticles

**Figure 3:**
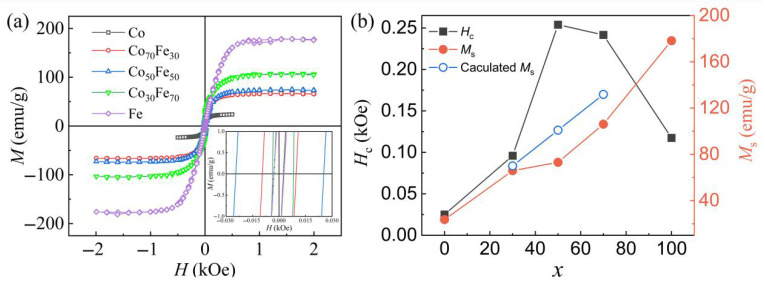
Room-temperature hysteresis loops of amorphous Co_100−x_Fe_x_ measured by VSM (**a**) and the dependence of *M*_s_ and *H*_c_ on *x* (**b**).

The typical hysteresis loops of Co_100−x_Fe_x_ measured by VSM are displayed in Figure 3a. It is clear that with increasing Fe content, the saturation magnetization (*M*_s_) increases. The measured *M*_s_ values are 23.6, 65.8, 73.1, 106.1, and 178.2 emu/g for Co, Co_70_Fe_30_, Co_50_Fe_50_, Co_30_Fe_70_, and Fe, respectively. Based on the volume fractions and the corresponding *M*_s_ values of pure Co and Fe [15], the calculated *M*_s_ values for Co_70_Fe_30_, Co_50_Fe_50_, and Co_30_Fe_70_ are 70, 100.9, and 131.8 emu/g, respectively, showing an increasing trend with Fe content, which is consistent with the experimental results presented in Figure 3b. However, the calculated *M*_s_ is slightly larger than the measured *M*_s_, which is because the effect of particle size was not considered in the calculation. For the crystallized Co_100−x_Fe_x_ particles or bulk materials [17,29,33], Co_3_Fe_7_ always exhibits the highest *M*_s_. In contrast, the *M*_s_ of the amorphous Co_100−x_Fe_x_ shows relatively smaller particle size, which may be attributed to the lack of long-range order, crystallinity, or oxidation. From Figure 3b, the coercive field (*H*_c_) first increases from 26 to 250 Oe as the Fe content increases from 0 to 50 and then decreases to 117 Oe when *x* = 100. For Co_100−x_Fe_x_, if only the influence of atomic ratio is considered, then the coercivity will increase as *x* increases, as Fe exhibits higher coercivity than Co. However, the coercivity decreases when *x* larger than 50. The relatively low coercivity of Fe may be due to the influence of size effect. Meanwhile, the large specific surface area, which is conducive to the formation of an oxide layer on the surface, might also lead to a higher coercivity for Co_50_Fe_50_ and Co_30_Fe_70_ as compared with Fe. Therefore, Fe is considered the optimal absorbent due to its higher *M*_s_ and lower *H*_c_. It is well known that the absorbing properties of the magnetic loss-type microwave absorption materials are highly influenced by the magnetization property. In order to achieve strong magnetic loss, a high magnetization value of the magnetic materials is required [10,34]. Thus, the magnetic loss should increase with the increase in Fe content in Co_100−x_Fe_x_ due to the dependence of *M*_s_ on the Fe content.

### 3.3. Microwave Performance Characterization of the Amorphous Co_100−x_Fe_x_ Nanoparticles

**Figure 4:**
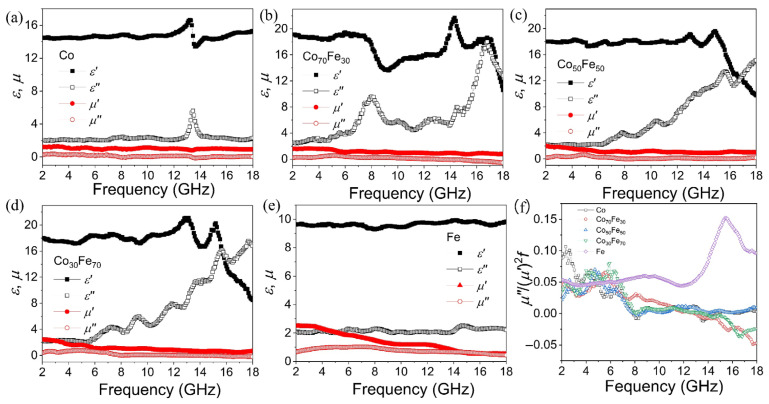
The complex permittivity and permeability of Co (**a**), Co_70_Fe_30_ (**b**), Co_50_Fe_50_ (**c**), Co_30_Fe_70_ (**d**), and Fe (**e**) with frequency in the range of 2–18 GHz. Frequency dependence of *μ*″(*μ*′)^−1^*f*^−1^ values of Co_100−x_Fe_x_ (**f**).

Generally, large *ε*′ and low *μ*′ are the primary obstacles to achieving good impedance matching in the magnetic metal materials, making the reduction in *ε*′ a key focus [35]. According to the free electron-scattering theory [36], higher resistivity corresponds to lower permittivity. The increased resistivity in amorphous metals, caused by internal disorder, leads to reduced electrical conductivity. This phenomenon may enhance impedance-matching characteristics. Figure 4 demonstrates the frequency-dependent complex permittivity and permeability of amorphous Co_100−x_Fe_x_ nanoparticles across the 2–18 GHz frequency range. The complex permittivity of amorphous Co demonstrates *ε*′ ≈ 14.5 and *ε*″ ≈ 2, both significantly lower than those of crystalline cobalt [29]. Additionally, a distinct formant peak can be observed at approximately 13.5 GHz in Figure 4a. The dielectric constant of amorphous Fe is lower than that of Co, Co_70_Fe_30_, Co_50_Fe_50_, and Co_30_Fe_70_. This difference may arise from the size effect. However, for Co, Co_70_Fe_30_, Co_50_Fe_50_, and Co_30_Fe_70_ with comparable dimensions, the dielectric constants exhibit similar magnitudes but distinct dielectric loss peaks. Therefore, the Co/Fe ratio primarily governs the dielectric characteristics in these materials. In Co_70_Fe_30_, Co_50_Fe_50_, and Co_30_Fe_70_ nanoparticles, multiple resonance peaks can be observed in the dielectric spectra. As shown in Figure 4b–d, the value of *ε*′ fluctuates around 18 when the frequency is below 16 GHz and decreases to 8 as the frequency increases from 16 to 18 GHz. The value of *ε*″ increases from 2 to 16 as the frequency increases from 2 to 18 GHz, as shown in Figure 4b–d. Notably, pure Fe maintains stable dielectric characteristics with *ε*′ ≈ 9.8 and *ε*″ ≈ 2 throughout this frequency range in Figure 4e. These results confirm that amorphous Co_100−x_Fe_x_ alloys demonstrate substantially lower *ε*′ values as compared with their crystalline counterparts [29]. Resonance behaviors in permittivity generally originate from space–charge polarization, dipole polarization, ionic polarization, and electronic polarization [37]. However, because ionic and electronic polarizations occur at THz and PHz frequencies, respectively, dipole polarization becomes the dominant mechanism over space–charge polarization in metal-based composites at higher frequencies (typically in the GHz range) [38]. Therefore, the permittivity resonance observed in the metal–paraffin mixtures arises predominantly from dipole polarization. According to the Debye relaxation model [39], the Cole–Cole curves (Figure S1 and Table S2, Supplementary Materials) indicate that, for the Co, Co_70_Fe_30_, Co_50_Fe_50_, and Co_30_Fe_70_ composites, losses beyond dipole polarization contribute to the permittivity spectra. These additional contributions likely include conductance loss and interfacial polarization between Co, Fe, carbon, and paraffin. In contrast, the Cole–Cole plot for amorphous Fe deviates from the characteristic semicircular shape, demonstrating the absence of a distinct dielectric relaxation-loss peak within the measured frequency range.

The *μ*′ and *μ*″ of the complex permeability spectra for amorphous Co_100−x_Fe_x_ are shown in Figure 4a–e. The *μ*_r_ is significantly lower than *ε*_r_, and several small peaks can be observed in the samples. As the frequency increases from 2 to 18 GHz, the values of *μ*′ and *μ*″ are mainly distributed in the ranges of 1.25 to 0.75 and 0.5 to 0, respectively, for Co, Co_70_Fe_30_, Co_50_Fe_50_, and Co_30_Fe_70_, as shown in Figure 4a–d. The permeability characteristics of Fe exhibit distinct frequency dependence in Figure 4e, with *μ*′ decreasing from 2.5 to 0.5 and *μ*″ diminishing from 1.1 to 0.5 across the measured spectrum. The μ″ spectrum demonstrates a broad magnetic resonance spanning from 2–9.5 GHz, reaching maximum dissipation (*μ*″ = 1.1) at 5 GHz. These magnetic loss mechanisms can be attributed to multiple contributions: hysteresis losses, domain-wall resonance, eddy current losses, exchange resonance, and natural resonance [15,17]. The low-amplitude microwave field conditions render hysteresis losses and domain-wall resonance contributions negligible. Figure 4f demonstrates the characteristic frequency dependence of *μ*″(*μ*′)^−1^*f*^−1^ values across all samples. According to the classical eddy current theory, frequency-independent *μ*″(*μ*′)^−1^*f*^−1^ curves would indicate dominant eddy current effects. However, the observed monotonic decrease in these normalized values with increasing frequency (from 0.12 to 0.04 GHz^−1^) confirms effective eddy current suppression in the amorphous structure. In this case, hysteresis loss and domain-wall resonance can be excluded due to the weakly applied microwave field. As shown in Figure 4f, the frequency dependence of the *μ*″(*μ*′)^−1^*f*^−1^ values for all samples varies with frequency, indicating that the eddy current effect can also be excluded [40,41]. According to the Landau–Lifshitz-Gilbert equation [42], the real part and the imaginary part of the magnetic permeability of the amorphous Fe has been fitted with three format peaks, as shown in Figure S1 and Table S2 (Supplementary Materials). The peaks at 4.7 GHz and 13.2 GHz are attributed to exchange resonance based on Aharoni’s theory [43,44], while the peak at 5.3 GHz corresponds to natural resonance according to the natural resonance theory [45]. Therefore, exchange and natural resonance may play the dominant role in the magnetic loss mechanism. Meanwhile, superior magnetic loss can result in strong microwave attenuation. However, compared with crystallized Co_100−x_Fe_x_ alloys, the *μ*′ and *μ*″ values of the amorphous Co_100−x_Fe_x_ alloys are slightly lower, which may be disadvantageous for magnetic loss. Nevertheless, considering impedance matching, the reduction in permittivity may be more crucial than the lower permeability for Co_100−x_Fe_x_.

**Figure 5:**
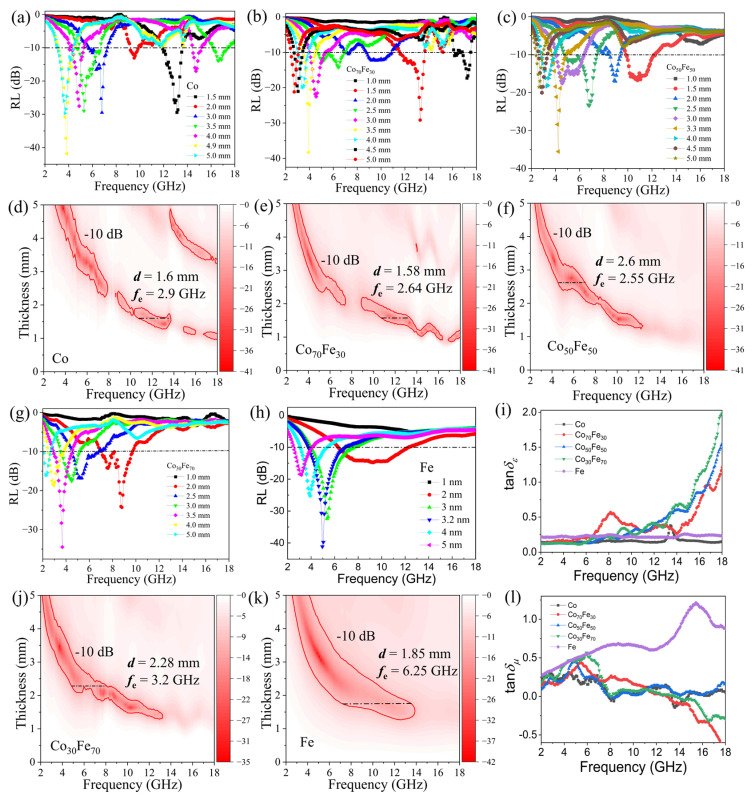
Calculated reflection loss spectra with different thicknesses of Co (**a**,**d**), Co_70_Fe_30_ (**b**,**e**), Co_50_Fe_50_ (**c**,**f**), Co_30_Fe_70_ (**g**,**j**), and Fe (**h**,**k**) in the frequency range of 2–18 GHz. Dielectric loss tangent (**i**) and magnetic loss tangent (**l**) of Co_100−x_Fe_x_.

Based on transmission line theory, the frequency dependence of the reflection loss (RL) of Co_100−x_Fe_x_ is calculated using the relative permeability, permittivity, and absorber thickness, according to the following equations [46,47]:

(1) $$Z_{in}=Z_{0}\sqrt{\frac{\mu_{r}}{\varepsilon_{r}}}tanh\left[j\frac{2\pi df}{c}\sqrt{\mu_{r}\varepsilon_{r}}\right]$$

(2) $$RL=20log\frac{\left|Z_{in}-Z_{0}\right|}{\left|Z_{in}+Z_{0}\right|}$$

where $Z_{0}=\sqrt{\mu_{0}/\varepsilon_{0}}$ is the impedance of free space, *Z*_in_ is the input impedance, *c* is the velocity of electromagnetic waves in free space, *f* is the frequency of microwaves, and *d* is the thickness of the composite. Figure 5 presents the calculated reflection loss (RL) spectra of Co, Co_70_Fe_30_, Co_50_Fe_50_, Co_30_Fe_70_, and Fe at various thicknesses. The optimal RL values reach 41.8, 38.6, 35.6,34.6, and 42 dB at 3.85, 3.93 4.25, and 3.68, and 5.02 GHz for Co, Co_70_Fe_30_, Co_50_Fe_50_, Co_30_Fe_70_, and Fe, respectively, with corresponding matching thicknesses of 4.9, 3.5, 3.3, 3.5, and 3.2 mm. Additionally, the absorption frequency range where the reflection loss is below −10 dB corresponds to at least a 90% attenuation of the incident microwave energy. As seen in Figure 5d–f,j,k, the effective absorption bandwidth (EAB) corresponding to the RL values below −10 dB for Co, Co_70_Fe_30_, Co_50_Fe_50_, Co_30_Fe_70_, and Fe are 2.9, 2.64, 2.55, 3.2, and 6.25 GHz, respectively. These bandwidths occur within the frequency ranges of 10.8–13.7 GHz, 10.67–13.31 GHz, 4.68–7.49 GHz, 4.84–8.04 GHz, and 6.56–12.12 GHz at absorber thicknesses of 1.6, 1.58, 2.55, 2.28, and 1.85 mm, respectively. These results demonstrate the excellent microwave absorption properties of the amorphous Co_100−x_Fe_x_ nanoparticles. To investigate the mechanism of microwave absorption, the dielectric loss tangent ($tan\delta_{\varepsilon }=\varepsilon \prime \prime /\varepsilon \prime$ and the magnetic loss tangent ($tan\delta_{\mu }=\mu \prime \prime /\mu \prime$) of amorphous Co_100−x_Fe_x_ were calculated. As shown in Figure 5i, the tan δ_ₑ_ of Co exhibits a peak value of 0.25 at approximately 13.5 GHz. For Co_70_Fe_30_, Co_50_Fe_50_, and Co_30_Fe_70_, the $tan\delta_{\varepsilon }$ increases from nearly 0 to 2.0 as the frequency rises from 10 to 18 GHz. In contrast, for Fe, the $tan\delta_{\varepsilon }$ remains almost constant across the frequency range. The stable dielectric loss below 10 GHz indicates a well-balanced electromagnetic wave impedance-matching in the composites [48]. As shown in Figure 5 the $tan\delta_{\mu }$ of Co, Co_70_Fe_30_, Co_50_Fe_50_, and Co_30_Fe_70_ exhibits several weak peaks in the 2–8 GHz range and fluctuates from around zero to 8 and 18 GHz. For Fe, the $tan\delta_{\mu }$ shows two distinct peaks spanning the ranges of 2–12 GHz and 12–18 GHz. These results suggest that dielectric loss is the primary contributor to microwave absorption in Co_70_Fe_30_, Co_50_Fe_50_, and Co_30_Fe_70_ at frequencies above 9 GHz. However, the RL performance in the 9–18 GHz range for these samples is suboptimal, likely due to impedance mismatch. In contrast, magnetic loss dominates in the 2–8 GHz range, contributing to the superior RL values observed in this frequency region.

**Figure 6:**
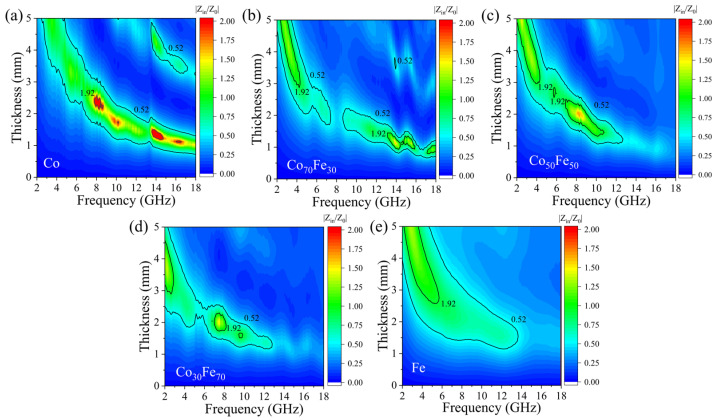
Impedance matching (|Z_in_/Z_0_|) of Co (**a**), Co_70_Fe_30_ (**b**), Co_50_Fe_50_ (**c**), Co_30_Fe_70_ (**d**), and Fe (**e**).

According to the measured data of the electromagnetic parameters, the input impedance Z_in_/Z_0_ of the absorber is given in Equation (2). Normally, when the value of Z_in_/Z_0_ approaches 1, an ideal impedance match can be achieved, which means the EM wave enters the absorber with zero reflection on the absorber–air contact interfaces [49]. If the attenuation property is not the limiting factor for microwave absorption when RL is less than −10 dB [50], as shown below,

(3) $$RL=20log\frac{\left|Z_{in}-Z_{0}\right|}{\left|Z_{in}+Z_{0}\right|}\leq -10,$$

the corresponding area of the EAB is

(4) $$0.52\approx \frac{\sqrt{10}-1}{\sqrt{10}+1}\leq \left|\frac{Z_{in}}{Z_{0}}\right|\leq \frac{\sqrt{10}+1}{\sqrt{10}-1}\approx 1.92$$

which can be denoted as the impedance-matching area. The contour maps of the calculated impedance match (|Z_in_/Z_0_|) for Co (a), Co_70_Fe_30_ (b), Co_50_Fe_50_ (c), Co_30_Fe_70_ (d), and Fe (e) can be found in Figure 6. It is obvious that the impedance-matching area of the amorphous Fe is better than others, which is a good explanation for amorphous Fe showing the best microwave absorption performance of all the samples. However, when the frequency of the microwave is larger than 14 GHz, the impedance matching deteriorates, indicating poor microwave-absorption performance in amorphous Fe at these frequencies. For amorphous Co, the impedance-matching area also demonstrates good values and is distributed from 2.7 to 18 GHz. Co_70_Fe_30_, Co_50_Fe_50_, and Co_30_Fe_70_ show inferior impedance matching because of their small impedance-matching areas, as seen in Figure 6b–d. It is evident that in the amorphous state, the ratio of Co–Fe is not a successful approach for optimizing impedance matching and enhancing microwave absorption performance.

Table 1 compares the microwave absorption properties of several CoFe-based systems with this work. Although these materials exhibit excellent absorption performances, particularly composites and those with unique geometries, their syntheses typically involves complex procedures or extended calcination times. In this work, amorphous CoFe was synthesized via a simple one-step aqueous reduction method. Although the minimum RL values of amorphous Co_100−x_Fe_x_ is in the moderate range, their low material densities and straightforward synthesis routes maintain competitive viability for microwave absorption applications.

## 4. Conclusions

In summary, amorphous CoFe nanoparticles with different atomic ratios were successfully synthesized by a simple aqueous reduction at room temperature. The amorphous state of Co_100−x_Fe_x_ was confirmed by XRD and TEM. The atomic ratio plays a crucial role in the magnetic properties, which also have a great effect on the dielectric constant and the values of impedance matching. Remarkably, the synthesized amorphous Co_100−x_Fe_x_ nanoparticles exhibit an excellent electromagnetic wave absorption performance, demonstrating a broad effective absorption bandwidth of 6.25 GHz, an ultra-thin matching thickness of 1.85 mm, and a strong reflection loss of −42 dB.

## Figures and Tables

**Table 1:** Reflection loss information of similar absorbers.

Sample	State	RL (dB)	*f*_r_ (GHz)	*d*_O_ (mm)	EAB (GHz)	*d*_EAB_ (mm)	Filled Mass Ratio	Ref.
Fe_7_Co_3_ flower	Crystalline	−53.6	14.3	1.55	6.8	2.0	7:3	[29]
Fe nanoparticle	Amorphous	−53.2	6.4	2.4	3.9	1.5–5.0	4:1	[14]
CoFe nanowires	Amorphous	−25.9	4	3	5.4	1–5	4:1	[51]
Hexagonal-cone like Fe_50_Co_50_	Crystalline	−22	10.4	1.5	7.1	1.5	7:3	[52]
FeCo@TiO_2_ material @PPy-M16	Crystalline	−29.8	/	1.9	10.21	2.63	1:1	[53]
Co	Amorphous	−41.8	3.85	4.9	2.9	1.6	1:1	This work
Co_70_Fe_30_	Amorphous	−38.6	3.93	3.5	2.64	1.58	1:1	This work
Co_50_Fe_50_	Amorphous	−35.6	4.25	3.3	2.55	2.55	1:1	This work
Co_30_Fe_70_	Amorphous	34.6	3.65	3.5	2.28	2.28	1:1	This work
Fe	Amorphous	42	5.02	3.2	6.25	1.85	1:1	This work

*f*_r_ and *d*_O_ stand for the optimal absorption peak position and the corresponding matching thickness, respectively. *d*_EAB_ is the thickness corresponding to the EAB.

## Data Availability

The data provided in this study are available from the corresponding author.

## Supplementary Materials

The following supporting information can be downloaded at: https://www.mdpi.com/article/10.3390/nano15141091/s1, Table S1. The element composition of the electrocatalysts obtained by ICP measurements. Table S2 Fitting parameters for permeability dispersion spectra. Figure S1 The fitted spectra of the real part (a) and imaginary part (b) of the magnetic permeability of amorphous Fe. Figure S2 The Cole-Cole curve of Co, Co_70_Fe_30_, Co_50_Fe_50_, Co_30_Fe_70_ and Fe.

## References

[1] Liu Y., Wei X., He X., Yao J., Tan R., Chen P., Yao B., Zhou J., Yao Z. (2023). Multifunctional Shape Memory Composites for Joule Heating, Self-Healing, and Highly Efficient Microwave Absorption. Adv. Funct. Mater..

[2] Li F., Zhan W., Su Y., Siyal S.H., Bai G., Xiao W., Zhou A., Sui G., Yang X. (2020). Achieving Excellent Electromagnetic Wave Absorption of ZnFe_2_O_4_@CNT/Polyvinylidene Fluoride Flexible Composite Membranes by Adjusting Processing Conditions. Compos. Part A Appl. Sci. Manuf..

[3] Wang Y., Li X., Han X., Xu P., Cui L., Zhao H., Liu D., Wang F., Du Y. (2020). Ternary Mo_2_C/Co/C Composites with Enhanced Electromagnetic Waves Absorption. Chem. Eng. J..

[4] Cao M.S., Wang X.X., Zhang M., Cao W.Q., Fang X.Y., Yuan J. (2020). Variable-Temperature Electron Transport and Dipole Polarization Turning Flexible Multifunctional Microsensor beyond Electrical and Optical Energy. Adv. Mater..

[5] Wang X.I., Cao W.I., Cao M.H., Yuan J. (2020). Assembling Nano–Microarchitecture for Electromagnetic Absorbers and Smart Devices. Adv. Mater..

[6] Hong Ng V.M., Huang H., Zhou K., Lee P.S., Que W., Xu J.Z., Kong L.B. (2017). Recent Progress in Layered Transition Metal Carbides and/or Nitrides (MXenes) and Their Composites: Synthesis and Applications. J. Mater. Chem. A.

[7] Pan F., Ning M., Li Z., Batalu D., Guo H., Wang X., Wu H., Lu W. (2023). Sequential Architecture Induced Strange Dielectric-Magnetic Behaviors in Ferromagnetic Microwave Absorber. Adv. Funct. Mater..

[8] Liang H., Hui S., Zhang L., Tao K., Chen Q., Lu W., Wu H. (2025). High-Density Dual Atoms Pairs Coupling for Efficient Electromagnetic Wave Absorbers. Small.

[9] Xu C., Liu P., Wu Z., Zhang H., Zhang R., Zhang C., Wang L., Wang L., Yang B., Yang Z. (2022). Customizing Heterointerfaces in Multilevel Hollow Architecture Constructed by Magnetic Spindle Arrays Using the Polymerizing-Etching Strategy for Boosting Microwave Absorption. Adv. Sci..

[10] Jian X., Wu B., Wei Y., Dou S., Mahmood N. (2016). Facile Synthesis of Fe_3_O_4_/GCs Composites and Their Enhanced Microwave Absorption Properties. ACS Appl. Mater. Interfaces.

[11] Su G.Y., You M.C., Chuang K.W., Wu M.H., Hsieh C.H., Lin C.Y., Yang C.Y., Anbalagan A.K., Lee C.H. (2023). Investigating Anisotropic Magnetoresistance in Epitaxially Strained CoFe Thin Films on a Flexible Mica. Nanomaterials.

[12] Cheng J.C., You M.C., Anbalagan A.K., Su G.Y., Chuang K.W., Yang C.Y., Lee C.H. (2025). Investigation of a magnetic sensor based on the magnetic hysteresis loop and anisotropic magnetoresistance of cofe thin films epitaxial grown on flexible mica and rigid mgo substrates with strain effect. Micromachines.

[13] Miao Y., Yang D., Jia L., Li X., Yang S., Gao C., Xue D. (2021). Magnetocrystalline anisotropy correlated negative anisotropic magnetoresistance in epitaxial fe30co70 thin films. Appl. Phys. Lett..

[14] Wang Z., Zuo Y., Yao Y., Xi L., Du J., Wang J., Xue D. (2013). Microwave Absorption Properties of Amorphous Iron Nanostructures Fabricated by a High-Yield Method. J. Phys. D Appl. Phys..

[15] Xi L., Wang Z., Zuo Y., Shi X. (2011). The Enhanced Microwave Absorption Property of CoFe_2_O_4_ Nanoparticles Coated with a Co_3_Fe_7_-Co Nanoshell by Thermal Reduction. Nanotechnology.

[16] Sun Y., Zhang J., Zong Y., Deng X., Zhao H., Feng J., He M., Li X.H., Peng Y., Zheng X. (2019). Crystalline-Amorphous Permalloy@Iron Oxide Core-Shell Nanoparticles Decorated on Graphene as High-Efficiency, Lightweight and Hydrophobic Microwave Absorbents. ACS Appl. Mater. Interfaces.

[17] Yan L., Wang J., Han X., Ren Y., Liu Q., Li F. (2010). Enhanced Microwave Absorption of Fe Nanoflakes after Coating with SiO_2_ Nanoshell. Nanotechnology.

[18] Deng Z., Li Y., Zhang H.B., Zhang Y., Luo J.Q., Liu L.X., Yu Z.Z. (2019). Lightweight Fe@C Hollow Microspheres with Tunable Cavity for Broadband Microwave Absorption. Compos. Part B Eng..

[19] Li X.P., Deng Z., Li Y., Zhang H.B., Zhao S., Zhang Y., Wu X.Y., Yu Z.Z. (2019). Controllable Synthesis of Hollow Microspheres with Fe@Carbon Dual-Shells for Broad Bandwidth Microwave Absorption. Carbon.

[20] Liu J.R., Itoh M., Terada M., Horikawa T., Machida K.I. (2007). Enhanced Electromagnetic Wave Absorption Properties of Fe Nanowires in Gigahertz Range. Appl. Phys. Lett..

[21] Li W., Lv J., Zhou X., Zheng J., Ying Y., Qiao L., Yu J., Che S. (2017). Enhanced and Broadband Microwave Absorption of Flake-Shaped Fe and FeNi Composite with Ba Ferrites. J. Magn. Magn. Mater..

[22] Wen S., Liu Y., Zhao X. (2015). Facile Chemical Synthesis, Electromagnetic Response, and Enhanced Microwave Absorption of Cobalt Powders with Controllable Morphologies. J. Chem. Phys..

[23] Wen S., Liu Y., Zhao X., Cheng J., Li H. (2014). Synthesis, Dual-Nonlinear Magnetic Resonance and Microwave Absorption Properties of Nanosheet Hierarchical Cobalt Particles. Phys. Chem. Chem. Phys..

[24] Yang Y., Xu C., Xia Y., Wang T., Li F. (2010). Synthesis and Microwave Absorption Properties of FeCo Nanoplates. J. Alloys Compd..

[25] Kvpm S., Prozorov R., Gedanken A. (1998). Sonochemical Preparation and Characterization of Nanosized Amorphous Co-Ni Alloy Powders. J. Mater. Chem..

[26] Suslick K.S., Choe S.B., Cichowlas A.A., Grinstaff M.W. (1991). Sonochemical Synthesis of Amorphous Iron. Nature.

[27] Carroll K.J., Hudgins D.M., Brown L.W., Yoon S.D., Heiman D., Harris V.G., Carpenter E.E. (2010). Annealing of Amorphous Fe_x_Co_100-x_ Nanoparticles Synthesized by a Modified Aqueous Reduction Using NaBH_4_. J. Appl. Phys..

[28] Athanassiou E.K., Grossmann P., Grass R.N., Stark W.J. (2007). Template Free, Large Scale Synthesis of Cobalt Nanowires Using Magnetic Fields for Alignment. Nanotechnology.

[29] Cheng Y., Ji G., Li Z., Lv H., Liu W., Zhao Y., Cao J., Du Y. (2017). Facile Synthesis of FeCo Alloys with Excellent Microwave Absorption in the Whole Ku-Band: Effect of Fe/Co Atomic Ratio. J. Alloys Compd..

[30] Lin T.C., Seshadri G., Kelber J.A. (1997). A Consistent Method for Quantitative XPS Peak Analysis of Thin Oxide Films on Clean Polycrystalline Iron Surfaces. Appl. Surf. Sci..

[31] Biesinger M.C., Payne B.P., Grosvenor A.P., Lau L.W.M., Gerson A.R., Smart R.S.C. (2011). Resolving Surface Chemical States in XPS Analysis of First Row Transition Metals, Oxides and Hydroxides: Cr, Mn, Fe, Co and Ni. Appl. Surf. Sci..

[32] Moulder J.F., Stickle W.F., Sobol P.E., Bomben K.D. (1992). Handbook of X-ray Photoelectron Spectroscopy.

[33] Grinstaff M.W., Salamon M.B., Suslick K.S. (1993). Magnetic Properties of Amorphous Iron. Phys. Rev. B.

[34] Lu B., Huang H., Dong X.L., Zhang X.F., Lei J.P., Sun J.P., Dong C. (2008). Influence of Alloy Components on Electromagnetic Characteristics of Core/Shell-Type Fe–Ni Nanoparticles. J. Appl. Phys..

[35] Wang Y., Guan H., Du S., Wang Y. (2015). A Facile Hydrothermal Synthesis of MnO_2_ Nanorod–Reduced Graphene Oxide Nanocomposites Possessing Excellent Microwave Absorption Properties. RSC Adv..

[36] Ramo S., Whinnery J.R., Van Duzer T. (1994). Fields and Waves in Communication Electronics.

[37] Lacrevaz T., Fléchet B., Farcy A., Torres J., Gros-Jean M., Bermond C., Vo T., Cueto O., Blampey B., Angénieux G. (2006). Wide band frequency and in situ characterisation of high permittivity insulators (high k) for h.f. integrated passives. Microelectron. Eng..

[38] Ravindran R., Gangopadhyay K., Gangopadhyay S., Mehta N., Biswas N. (2006). Permittivity enhancement of aluminum oxide thin films with the addition of silver nanoparticles. Appl. Phys. Lett..

[39] Frenkel J., Doefman J. (1930). Spontaneous and induced magnetisation in ferromagnetic bodies. Nature.

[40] Wu M., Zhang Y., Hui S., Xiao T., Ge S., Hines W., Budnick J., Taylor G. (2002). Microwave Magnetic Properties of Co_50_/(SiO_2_)_50_ Nanoparticles. Appl. Phys. Lett..

[41] Wen F., Yi H., Qiao L., Zheng H., Zhou D., Li F. (2008). Analyses on Double Resonance Behavior in Microwave Magnetic Permeability of Multiwalled Carbon Nanotube Composites Containing Ni Catalyst. Appl. Phys. Lett..

[42] Deng L.J., Zhou P.H., Xie J.L., Zhang L. (2007). Characterization and microwave resonance in nanocrystalline FeCoNi flake composite. J. Appl. Phys..

[43] Amikam A. (1991). Exchange resonance modes in a ferromagnetic sphere. J. Appl. Phys..

[44] Aharoni A. (1997). Effect of surface anisotropy on the exchange resonance modes. J. Appl. Phys..

[45] Kittel C. (1948). On the Theory of Ferromagnetic Resonance Absorption. Phys. Rev..

[46] Naito Y., Suetake K. (1971). Application of Ferrite to Electromagnetic Wave Absorber and its Characteristics. IEEE Trans. Microw. Theory Tech..

[47] Liu J.R., Itoh M., Machida K.I. (2003). Electromagnetic Wave Absorption Properties of α-Fe/Fe_3_B/Y_2_O_3_ Nanocomposites in Gigahertz Range. Appl. Phys. Lett..

[48] Zhang X., Dong X., Huang H., Lv B., Lei J., Choi C. (2007). Microstructure and Microwave Absorption Properties of Carbon-Coated Iron Nanocapsules. J. Phys. D Appl. Phys..

[49] Wang L., Zhang J., Wang M., Che R. (2019). Hollow porous Fe_2_O_3_ microspheres wrapped by reduced graphene oxides with high-performance microwave absorption. J. Mater. Chem. C.

[50] Zhu T., Sun Y., Wang Y., Xing H., Zong Y., Ren Z., Yu H., Zheng X. (2021). Controllable synthesis of mof-derived fexni1x@c composites with dielectric–magnetic synergy toward optimized impedance matching and outstanding microwave absorption. J. Mater. Sci..

[51] Shen J., Yao Y., Liu Y., Leng J. (2019). Amorphous bimetallic nanowires with high-performance microwave absorption: A case for FeCo nanowires. Nano.

[52] Lv H., Ji G., Wang M., Shang C., Zhang H., Du Y. (2014). Hexagonal-cone like of Fe_50_Co_50_ with broad frequency microwave absorption: Effect of ultrasonic irradiation time. J. Alloys Compd..

[53] Zhang Y., Wang X., Dong T., Yang S., Yu Q., Yu B., Cai M., Zhou F. (2025). The synergistic enhancement of microwave absorption performance and corrosion resistance of FeCo by polypyrrole-M16 and TiO_2_. J. Colloid. Interf. Sci..

