# Supplementary material for: Enhanced Microwave Absorption Performance of Amorphous Co100−xFex Nanoparticles
Source: Nanomaterials (Basel). 2025 Jul 14;15(14):1091. doi: 10.3390/nano15141091 (PMC12299870; doi:10.3390/nano15141091)
Supplement: Supplementary file 1 [file nanomaterials-15-01091-s001.zip › nanomaterials-3682985-supplementary.pdf]

Supplementary Materials

Enhanced Microwave Absorption Performance of Amorphous $Co_{100-x}Fe_x$ Nanoparticles

Zhen Wang [1,*], Chao An [2], Fenglong Wang [1], Hongsheng Liang [1], Zhaoyang Hou [1], Hao Shen [1,*] and Hongjing Wu [3]

[1] Department of Applied Physics, School of Science, Chang'an University, Xi'an 710064, China; wangfenglong@chd.edu.cn (F.W.); hongshengliang@chd.edu.cn (H.L.); houzy@chd.edu.cn (Z.H.)
[2] School of Intelligent Manufacturing Engineering, Chongqing University of Arts and Sciences, Chongqing 402160, China; anchao@cqwu.edu.cn
[3] MOE Key Laboratory of Material Physics and Chemistry Under Extraordinary, School of Physical Science and Technology, Northwestern Polytechnical University, Xi'an 710072, China; wuhongjing@nwpu.edu.cn
* Correspondence: wangzhen@chd.edu.cn (Z.W.); shenhao@chd.edu.cn (H.S.)

Table S1. The element composition of the electrocatalysts obtained by ICP measurements.

Sample	Co content (mg/g)	Fe content (mg/g)	n_{Co}/n_{Fe}
$Co_{70}Fe_{30}$	584.91±5.25	248.24±2.89	2.23
$Co_{50}Fe_{50}$	278.42±4.52	237.34±3.27	1.11
$Co_{30}Fe_{70}$	221.26±3.67	639.50±3.51	0.33

Table S2 Fitting parameters for permeability dispersion spectra.

Peak	α	I	f (GHz)
P_1	0.75	0.6	4.7
P_2	0.45	0.44	8.23
P_3	0.2	0.09	13.2

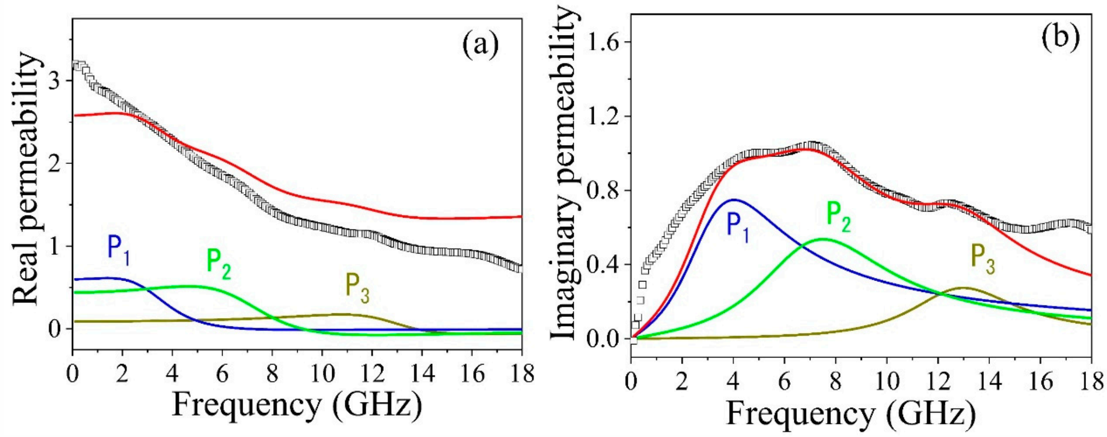

Figure S1 The fitted spectra of the real part (a) and imaginary part (b) of the magnetic permeability of amorphous Fe.

To understand such resonance behavior, the Landau–Lifshitz-Gilbert equation can be used to fit the dynamic permeability of magnetic materials [1].

$$\mu' = \mathrm{B} + \sum_{i}^{3} I_i \frac{[1-\left(\frac{f}{f_i}\right)^2(1-\alpha_i^2)]}{[1-\left(\frac{f}{f_i}\right)^2(1+\alpha_i^2)]^2 + 4\alpha_i^2\left(\frac{f}{f_i}\right)^2}$$

$$\mu'' = \sum_{i}^{3} I_i \frac{\left(\frac{f}{f_i}\right)\alpha_i[1+\left(\frac{f}{f_i}\right)^2(1-\alpha_i^2)]}{[1-\left(\frac{f}{f_i}\right)^2(1+\alpha_i^2)]^2 + 4\alpha_i^2\left(\frac{f}{f_i}\right)^2}$$

where f is the frequency, f_i is the spin resonance frequency, α_i is the damping constant, and I_i is the intensity of the band. In our experiment, the μ'' spectra should be a broad resonance band, and it can be phenomenologically analyzed to have three overlapped resonance peaks. We take amorphous Fe as an example. From Figure S1, the magnetic spectrum could be fitted by three formant peaks and the relevant fitting parameters are shown in Table S2.

The exchange resonance occurs at a higher resonance frequency than natural resonance, and they are potentially observable with samples having at least one small dimension. According to Aharoni's theory [2], $\omega/\gamma_0 = H_a + \frac{2A\mu_{kn}^2}{D^2 M_s}$, where A is the exchange constant, the eigenvalue is the root of the differential of the spherical Bezier function, and D is the crystal size. The two first μ_{kn} roots are $\mu_{11} = 2.08$, $\mu_{12} = 3.34$ [2, 3]. The calculated values of resonance frequency are 4.97 and 12.66 GHz, which are in accordance with the fitted resonance frequency 4.7 and 13.2 GHz.

According to natural resonance theory [4], the natural resonance peak can be expressed as $f_r = \frac{\gamma_0}{2\pi} H_a$ Where $\gamma_0$is the magnetogyric ratio (28 GHz/T), H_a is the effective anisotropy field. The natural resonant frequency varies linearly with the effective anisotropic field (H_a), and H_a depends on the anisotropy of the magnetic crystal as well as the morphology, size of the magnetic particles and the interaction between the particles. For spherical samples, shape anisotropy can be ignored $H_a = \frac{2K}{M_s}$ where K is magneto-crystalline anisotropy constant and M_s is the saturation magnetization. For amorphous magnetic materials, it is very difficult to obtain its effective anisotropic field. For the shortrange order of the amorphous material, magnetocrystalline anisotropy constant K_1 should be replaced by the local anisotropy constant [J. Phys. D Appl. Phys. 2013, 46, 135002]. For Fe, it is around 5×10^4 J/m^3. Then, the calculated natural resonance frequency was approximately 5.3 GHz for Fe, which might be corresponding to 8.23. Thus, the magnetic loss mechanism includes exchange and natural resonance.

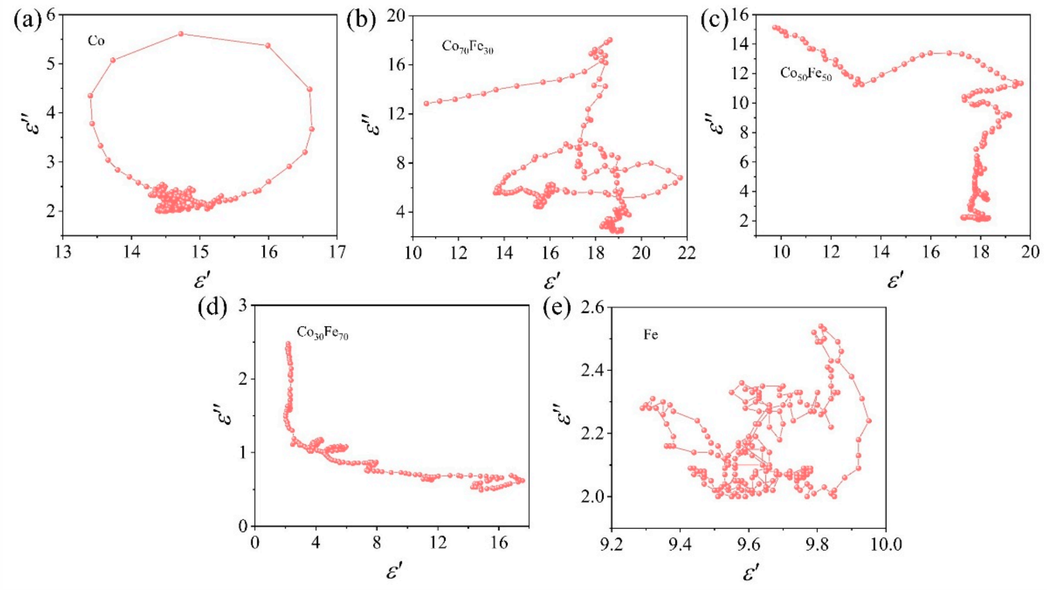

Figure S2 The Cole-Cole curve of Co, $Co_{70}Fe_{30}$, $Co_{50}Fe_{50}$, $Co_{30}Fe_{70}$ and Fe.

In general, the resonance behaviors of the permittivity originate from space-charge polarization, dipole polarization, ionic polarization, and electronic polarization. Since ionic polarization and electronic polarization occur at frequencies around THz and PHz [6], respectively, dipole polarization becomes dominant over space-charge polarization at higher frequencies in metal-based composites [7]. Consequently, the resonance in the permittivity of the metal–paraffin mixture should originate from dipole polarization. Debye dielectric relaxation model (Cole–Cole model) is adopted to demonstrate the dielectric loss mechanism of $Co_{100-x}Fe_x$. According to Debye relaxation equation, the relationship between ε′ and ε″ can be deduced [8]:

$$(\varepsilon' - \frac{\varepsilon_s + \varepsilon_\infty}{2})^2 + (\varepsilon'')^2 = (\frac{\varepsilon_s - \varepsilon_\infty}{2})^2$$

where ε_s and ε_∞ are static permittivity and permittivity at infinite frequency, respectively. The plot of ε'' versus ε' would be a single semicircle, which is usually defined as the Cole–Cole semicircle. Theoretically, when only dipoles are polarized in the material, its Cole-Cole curve should be of the semi-loop type, that is, each semicircle corresponds to one dipole relaxation process. Figure S2 shows the Cole-Cole curve of Co, $Co_{70}Fe_{30}$, $Co_{50}Fe_{50}$, $Co_{30}Fe_{70}$ and Fe. A Cole–Cole semicircle and two partly semicircles are clearly observed in the curves of Co. This suggests that the permittivity is mainly contributed by the dipole polarization. For $Co_{70}Fe_{30}$, $Co_{50}Fe_{50}$ and $Co_{30}Fe_{70}$, show more distorted Cole–Cole semicircles suggesting that, except dipole polarization, other loss may contribute to the permittivity spectra besides dielectric relaxation, such as conductance loss, interfacial polarization among Co, Fe, carbon and paraffin. For amorphous Fe, the graph does not conform to the Cole-Cole semi-circle feature, indicating that there is no obvious relaxation dielectric loss peak within the test frequency range.

References:

[1] Deng, L.J.; Zhou, P.H.; Xie, J.L.; Zhang, L. Characterization and microwave resonance in nanocrystalline FeCoNi flake composite. *Journal of Applied Physics,* 2007, *101,* 10, 10F905.

[2] Aharoni, Amikam. Exchange resonance modes in a ferromagnetic sphere. *Journal of Applied Physics*, 1991,69, 11, 7762-7764.
[3] Amikam, Aharoni. Effect of surface anisotropy on the exchange resonance modes. *Journal of Applied Physics*, 1997, 81, 2, 830-830.
[4] Kittel, C. *Phys. Rev.* 1948, 73, 155
[5] Wang, Z.; Zuo, Y.; Yao, Y.; Xi, L.; Du, J.; Wang, J.; Xue, D. Microwave Absorption Properties of Amorphous Iron Nanostructures Fabricated by a High-Yield Method. *J. Phys. D Appl. Phys.* 2013, 46, 135002.
[6] Lacrevaz, T.; B. Fléchet, Farcy, A.; Torres, J.; Gros-Jean, M.; Bermond, C.; Vo, T.; Cueto, O.; Blampey, B.; Angénieux, G. Wide band frequency and in situ characterisation of high permittivity insulators (high k) for h.f. integrated passives. *Microelectronic Engineering*, 2006, 83, 11-12, 2184-2188.
[7] Ravindran, R.; Gangopadhyay, K.; Gangopadhyay, S.; Mehta, N.; Biswas, N. Permittivity enhancement of aluminum oxide thin films with the addition of silver nanoparticles. *Applied Physics Letters*, 2006, 89, 26, 5243-39.
[8] Frenkel, J.; Doefman, J. Spontaneous and induced magnetisation in ferromagnetic bodies. Nature, 1930, 126, 3173, 274-275.
